# Diagnostic accuracy, yield, and comparative effectiveness of whole-body computed tomography in blunt trauma: A protocol for systematic review and meta-analysis

**DOI:** 10.1097/MD.0000000000024205

**Published:** 2021-01-15

**Authors:** Kyohei Nagasawa, Mitsunaga Iwata, Takashi Nihashi, Teruhiko Terasawa

**Affiliations:** a Department of Emergency and General Internal Medicine, Fujita Health University School of Medicine, Toyoake, Aichi; b Department of Diagnostic and Interventional Radiology, Aichi Cancer Center Hospital, Nagoya; c Department of Radiology, National Center for Geriatrics and Gerontology, Obu; d Department of Radiology, Fujita Health University, School of Medicine, Toyoake, Aichi, Japan.

**Keywords:** blunt trauma, diagnostic yield, emergency department, incidental findings, meta-analysis, mortality, sensitivity and specificity, whole-body computed tomography

## Abstract

**Objectives:**

Controversies emerge over routine performances of whole-body computed tomography (WBCT) in patients with blunt polytrauma. The existing randomized and non-randomized evidence is inconclusive, and during observations of non-trauma, incidental findings, detected by WBCT, have left uncertainty regarding their consequences and optimal management. Additionally, previous meta-analyses have failed to address the limitations of primary studies and issues associated with incidental findings. Therefore, we planned a new systematic review to address these points.

**Methods:**

We will search the PubMed, EMBASE, and Cochrane Central databases from inception to December 31, 2020, with no language restriction and perform full-text evaluation of potentially relevant articles. We will include prospective and retrospective studies with a single-gate design that assessed diagnostic accuracy and/or yield of WBCT to detect traumatic injuries, and studies that assessed incidental findings detected by WBCT. Additionally, we will include randomized controlled trials and non-randomized comparative studies that assessed the effectiveness of WBCT against conventional care, including selective computed tomography (CT). Studies of patients of all ages with blunt traumatic injuries, assessed at an emergency department, will be included. Two reviewers will extract data and rate the study validity via standard quality assessment tools. The primary outcome of interest will be reduction in mortality. Our secondary outcomes will include diagnostic accuracy and yield, detection of incidental findings and clinical outcomes associated with these detections, and improvement in other non-mortality clinical outcomes. We will qualitatively assess study, patient, and intervention characteristics and clinical outcomes. If appropriate, we will perform random-effects model meta-analyses to obtain summary estimates. Finally, we will assess the certainty of evidence by the grading the quality of evidence and strength of recommendations.

**Ethics and dissemination:**

Ethics approval is not applicable, as this is a secondary analysis of publicly available data. The review results will be submitted for publication in peer-reviewed journals.

**Prospero registration:**

CRD42020187852.

## Abstract 2

Supplemental Digital Content is available in the text

## 1 Introduction

Injury is a major cause of health loss with approximately 4.5 million deaths globally in 2017.^[1]^ Traumatic injuries are commonly divided into 2 broad categories: penetrating injuries (caused by foreign objects that penetrate tissue) and blunt injuries (caused by direct and/or indirect force with or without body movement toward, away from, or with the impacting object).^[2]^ Although the incidence rates of penetrating injury vary by geographic region, injuries attributed to blunt trauma are the most common worldwide, and are typically caused by traffic collisions and falls^[3]^ with approximately 1.2 million and 0.7 million deaths, respectively.^[1]^

Multiple trauma, also referred to as major trauma or polytrauma, the term applied to patients with multiple traumatic injuries with high mortality rates, is conventionally defined by an Injury Severity Score (ISS) ≥16,^[4]^ and recently, as proposed in the new Berlin definition, using ≥2 injuries with an Abbreviated Injury Scale (AIS) ≥3 and at least one additional clinical variable that reflects pathological conditions.^[5]^ Since blunt injuries may not necessarily be externally visible, accurate and timely diagnosis of specific injuries plays a critical part in the management of blunt multiple trauma. Advanced Trauma Life Support (ATLS) recommends a standardized, structured approach to trauma patients.^[6]^ This approach comprises the primary survey of life-threatening injuries coupled with implementing simultaneous resuscitation based on the Airway (assessment and protection), Breathing (and ventilation assessment), Circulation (assessment), Disability (assessment), and Exposure (with environmental control) (ABCDE) approach and the secondary survey by systematically assessing the injured body parts and their specific treatments.^[6]^ In the primary survey, x-ray examination of the chest and pelvis using a portable device, and bedside ultrasound examination of the perihepatic space, perisplenic space, pericardium, and pelvis, also known as focused assessment with sonography for trauma (FAST), are recommended as the standard adjunct diagnostic imaging.^[6]^ In the secondary survey, computed tomography (CT) is the mainstay to identify specific injuries.^[6]^

Advances in medical imaging technology have introduced multi-detector CT in clinical practice. With its rapid scanning and reconstruction speed, coupled with high quality non-invasive imaging, several routines including upfront pan-scanning protocols, whole-body CT (WBCT) in early trauma evaluation were proposed.^[7]^ With installation of a modern, high-quality, high-speed CT scanner system, adjacent to or in the emergency department (ED), precise images, rapidly obtained through upfront pan-scanning, could theoretically provide a better evaluation of the injured sites and can lead to better management to life-threatening injuries in a timely manner even in hemodynamically unstable trauma patients with specific indications.^[8]^ Examples of the expected benefits include timely provision of surgical interventions, such as decompressive surgery for severe brain injuries with intracranial hypertension, and surgical interventions and/or interventional radiology (IVR) for bronchial, lung, and aortic injuries or hemothorax and hemoperitoneum.

Since the first observation of the usefulness of upfront, routine use of WBCT in comparison with conventional management, based on selective CT, was reported in 2007,^[9]^ subsequent observational studies^[10–18]^ and meta-analyses^[19–22]^ consistently reported that WBCT-based management strategies were associated with better clinical outcomes, including mortality, than conventional management. Consequently, WBCT has gained rapid and wide adoption in clinical practice.

However, uncertainties exist regarding the comparative effectiveness of WBCT-based management vs conventional care because the promising comparative data from the observational studies were not validated in a Dutch non-blinded randomized controlled trial (RCT), REACT-2, enrolling approximately 1100 patients with trauma of varied severity, including 36% non-multiple trauma patients.^[23]^ Although several intermediate outcomes, such as the time required to diagnose traumatic injuries, were improved, the effect of WBCT analyzed based on the intention-to-treat (ITT) analysis (i.e., the ITT effect)—the effect of assignment to the WBCT group—failed to show a mortality benefit. As expected, 46% of the patients in the conventional group ultimately underwent a WBCT after the primary survey; non-blinded treating personnel sequentially performed CT scans of all segmental body regions.^[23]^ Post-randomization confounding is a well-known consequence in pragmatic RCTs such as REACT-2 and needs special care^[24]^ However, the ITT effect may not always represent the ultimate target in RCTs; the per-protocol effect, an alternative to the ITT effect, which also accounts for adherence to the assigned interventions, may be of more relevance to patients and their clinicians.^[25]^ Nevertheless, a most recent meta-analysis^[26]^ that included REACT-2 performed naive synthesis methods, not rigorously addressing these methodological heterogeneities.

In addition to increased radiation exposure, WBCT necessarily detects incidental findings other than traumatic injuries, which include both benign and neoplastic lesions as well as vascular abnormalities, such as enlarged aneurysms.^[27]^ Clinical consequences of these findings, however, are uncertain and their optimal managements have not yet been established. For instance, a recent observation of a regional trauma registry in the USA involving 957 patients reported non-trauma abnormal findings in 40%.^[28]^ Nearly 90% of the CT-detected incidental findings were nontrivial and deemed to require additional medical interventions of various invasiveness, raising concerns at both individual and societal levels. Again, previous systematic reviews did not address the issues stemming from incidental findings.

Given that it has been over 4 years since the most recent meta-analysis report was published, and that there has been an absence of new, rigorously conducted systematic reviews that addressed the aforementioned limitations, we planned to conduct a new systematic review and meta-analysis. Our objectives are 3 fold: (a) an update of the comparative effectiveness of WBCT-based management compared with conventional imaging-based management, (b) qualitative and quantitative syntheses of the evidence on clinical outcomes associated with incidental findings of WBCT, and (c) comprehensive evidence review of predictors that identify in which subgroups WBCT is more useful or harmful.

## 2 Methods

**Figure 1:**
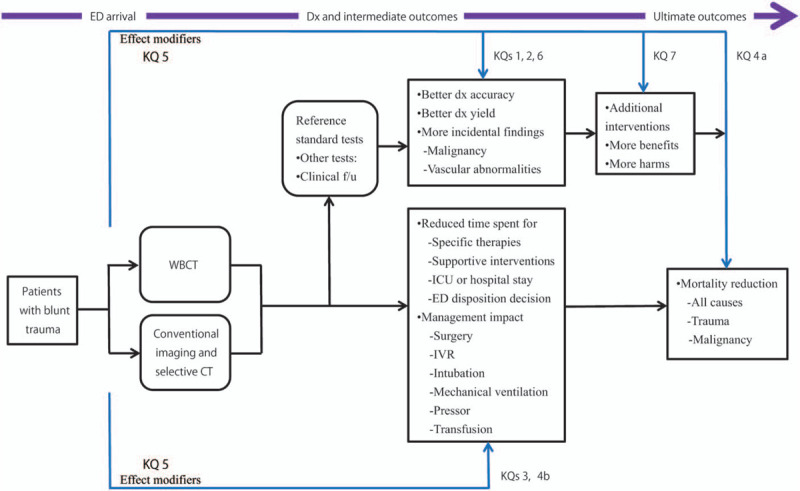
Analytic framework. CT = computed tomography, Dx = diagnosis, ED = emergency department, f/u = follow-up, ICU = intensive care unit, IVR = interventional radiology, KQ = key question, WBCT = whole-body computed tomography.

This systematic review and meta-analysis protocol follows the preferred reporting items for the systematic review and meta-analysis protocols 2015 statement (PRISMA-P) and in the event of protocol amendments, the date of each amendment will be accompanied by a description of the change and the rationale.^[29]^ Based on the established framework for assessing levels of clinical effectiveness of diagnostic tests^[30]^ and the analytic framework shown in Figure 1, we have formulated the following 7 key questions (KQs):

KQ 1 (diagnostic accuracy):

What is the diagnostic accuracy of WBCT to detect traumas?

a. All patients with blunt traumatic injuries (regardless of severity)
b. Patients with blunt multiple trauma only

KQ 2 (diagnostic impact):

What is the proportion of patients for whom WBCT is useful in diagnosing traumatic injuries in comparison with conventional imaging-based assessment?

a. All patients with blunt traumatic injuries (regardless of severity)
b. Patients with blunt multiple trauma only

KQ 3 (management decision impact):

How often does WBCT change diagnostic or therapeutic strategies in comparison with conventional imaging-based assessment?

a. Use of therapeutic interventions
b. Use of supportive interventions
c. Use of diagnostic interventions

KQ 4 (clinical outcomes):

What is the comparative effectiveness between WBCT-based management strategies and conventional strategies?

a. Mortality
b. Time spent for specific therapeutic or supportive interventions, and ICU or hospital stay
c. Time spent before ED disposition decision

KQ 5 (effect modifiers):

What factors modify the effectiveness measures listed under the KQ 4?

KQ 6 (incidental findings; see the operational definition section for the grades of incidental findings):

In what percentage of trauma patients does WBCT detect incidental findings?

a. Malignancy
b. Major findings (that may cause mortality)
c. Moderate findings (that may cause morbidity)

KQ 7 (additional interventions and clinical effectiveness or harm related to incidental findings):

What are the consequences related to incidental findings detected by WBCT?

a. Additional management related to incidental findings
b. Subsequent clinical outcomes related to incidental findings

### 2.1 Information sources and search strategies

We will search the PubMed, EMBASE, and Cochrane Central Register of Controlled Trials (CENTRAL) databases from the inception of data collection through December 31, 2020, using free text terms, such as “whole body CT” and “multiple trauma” and their synonyms. The complete search strategy and full list of databases are available in the supplementary file, http://links.lww.com/MD/F533. The electronic search results will be imported into EndNote X9 (Clarivate Analytics, Philadelphia, USA) and duplicate results will be removed. As additional searches, we will peruse the reference lists of previously reported systematic reviews and meta-analyses. No language restrictions will be set.

### 2.2 Eligibility criteria

Table 1 presents our detailed inclusion criteria, which follow a generally accepted framework to formulate systematic review questions comprising 6 key components: populations, interventions, comparator interventions, outcomes, timings, and settings listed under the patient, and intervention, comparator, outcome, timing, and setting (i.e., the so-called PICOTS) framework.^[31,32]^ We will include any prospective or retrospective cohort or cross-sectional studies with a single-gate design^[33]^ that included ≥30 patients of all ages with blunt trauma and assessed the outcomes relevant to KQs 1, 2, 3, 5, 6, and 7. Regarding KQs 3 and 4, both RCTs and non-randomized studies of interventions (NRSIs)—cohort studies, case-control studies, controlled before-and-after studies, interrupted-time-series studies, and “quasi-” randomized studies—of any size that assessed the comparative effectiveness of WBCT in comparison with conventional management involving selective CT, will be eligible. We will exclude conference abstracts, primary studies with the outcome data unextractable from the publication, and studies based on mathematical modeling approaches, such as decision model or cost-effectiveness studies. To deal with across-study variations and insufficient reporting in the inclusion criteria of trauma patients (e.g., mixed populations not limited to patients with blunt trauma but jointly assessing patients with blunt trauma and those with penetrating injury or gun-shot injury), we will exclude studies that explicitly included >5% of patients with trauma other than blunt trauma, or studies including >10% of patients with unspecified trauma unless subgroup data on patients with blunt trauma exclusively are extractable.

**Table 1:** Inclusion criteria and clinical outcomes of interest based on the PICOTS framework.

PICOTS	Specific details
Population	Male and female patients of all ages with blunt trauma
Intervention test	Routine, upfront use of whole-body CT
Comparator/reference standard tests	• Composite reference standards comprising any adopted tests ± clinical follow-up (for KQs 1, 2, 3, 5, 6, and 7)• Conventional management including selective CT (for KQ 4) - Screening with FAST and portable chest and pelvis X-ray - Other screening strategies
Outcomes	KQ 1: Sensitivity and specificity • All patients with blunt traumatic injuries (regardless of severity) • Patients with blunt multiple trauma only KQ 2: Diagnostic yield • All patients with blunt traumatic injuries (regardless of severity) • Patients with blunt multiple trauma onlyKQ 3: Additional use of interventions • Therapeutic interventions (e.g., surgery, interventional radiology) • Supportive interventions (e.g., intubation and mechanical ventilation, pressor, transfusion) • Diagnostic interventionsKQ 4: Clinical outcomes • Mortality (4a) • Time spent for specific therapeutic or supportive interventions, and ICU or hospital stay (4b) • Time spent before ED disposition decision (4b)KQ 5: Effect modifiers • CT protocol • Cause of trauma (road traffic injuries vs. falls) • Severity of included patients (by multiple trauma vs. not; and AIS > 3 vs. not by anatomical regions)KQ 6: Incidental findings • Malignancy • Major findings • Moderate findingsKQ 7: Additional interventions (a) and clinical effectiveness or harm related with incidental findings (b) • Additional interventions deemed necessary (a-1) • Additional interventions actually performed (a-2) • Benefits related with detection of incidental findings (b-1) • Harms associated with detection of incidental findings (b-2)
Timings	• KQs 1, 2, and 6: at diagnosis• KQ 3: at diagnosis, at 24 h, and during the hospital stay• KQs 4 and 5: at 24 h, during the hospital stay, and at 30 d• KQ 7: not specified *a priori*
Settings	• ACS level 1 trauma center or comparable institutions

ACS = American College of Surgeons, CT = computed tomography, ED = emergency department, FAST = focused assessment with sonography in trauma, KQ = key question, NRSI = non-randomized studies of intervention, PICOTS = population, intervention, comparator, outcome, timing, and setting, RCT = randomized controlled trial.

Two independent reviewers will double-screen abstracts using Abstrackr, a web-based software for citation screening (Center for Evidence Synthesis in Health, Brown University, Province, USA). We will then peruse all potentially eligible full-text articles that ≥1 reviewers screen in for eligibility. All non-English publications will be translated into English before full-text assessment. Any discrepant results will be resolved by consensus. We will employ adjudication by a third reviewer in case of unresolved discrepancies.

### 2.3 Data extraction

Data will be extracted by 2 reviewers. One primary reviewer will extract descriptive data, and ≥1 other reviewer will verify all extracted data. Two independent reviewers will double-extract any numerical data on the outcomes of interest. Disagreements will be resolved by consensus, and a third reviewer will adjudicate any unresolved discrepancies. We will contact the study authors for any missing or unresolved numerical data by email. We will send 2 additional email correspondences if no response is received by 2 weeks after the previous correspondence attempt.

We will extract study and patient characteristics (for all **KQs**), characteristics of WBCT (for all **KQs**), characteristics of alternative testing strategies (for comparative studies of **KQs 1**, **2**, **3**, **4**, **5**, and **6**), characteristics of reference standards (for **KQs 1** and **2**), and definitions of reported clinical outcomes (for **KQs 3**, **4**, **5**, **6**, and **7**) as descriptive data.

Study characteristics will include study identification (first author and year), study location (country, city), study period (enrollment year), study design, enrollment methods (consecutive or not), number of centers, clinical setting, and inclusion and exclusion criteria.

Patient characteristics will include the number of patients, average age (range), male sex (%), average vital sign parameters (ranges), category of injury (penetrating, if any, vs blunt trauma) (%), mechanisms of injury, average ISS (range), injured anatomical parts per 5 ISS-based separate body regions (i.e., head and neck, face, chest, abdomen, and pelvic ring) (%), and their respective average AIS (range) and injuries categorized as severe trauma as defined as AIS ≥3 (%), and patients categorized as having multiple trauma (%). Items to extract data for the mechanisms of injury will include road traffic injuries (%), falls (%), and self- or inter-personal violence (%). The average and range values to be extracted for vital sign parameters will include respiratory rate (RR) (per minutes), pulse (beats per minutes), systolic blood pressure (SBP) (mmHg), and Glasgow Coma Scale (GCS) score (points), or the Revised Trauma Score in aggregate (points). We will consider a patient with multiple trauma if an ISS is ≥16,^[4]^ or the new Berlin definition criteria for polytrauma is satisfied (i.e., ≥2injuries with an AIS ≥3 and at least 1 additional clinical variable that reflects pathological conditions^[5]^). We will also record data on mortality prediction models such as the trauma and injury severity score (TRISS)^[34]^ or its variants, if any, were reported.

Test characteristics of WBCT will include location of CT scanner, timing of CT scanning, anatomical parts and specifications of CT scanning, use of contrast materials, and interpreter of CT results and experience. Items to extract data for the CT specifications include field of view, slice thickness and interval, X-ray tube voltage (kV), automatic tube current modulation reported as mAs product, number of detectors, pitch factors including gantry rotation speed and beam collimation, image reconstruction algorithms, and volume of CT dose index (CTDI_vol_).

The characteristics of alternative testing strategies will include pre-selective CT primary work-up (e.g., X-ray and ultrasound examination of specific body parts), routine indication of CT for specific body regions, if any, body regions for which CT was ultimately performed (%), WBCT performed after primary work-up (%), and other specific post-primary work-up imaging tests. Items to extract data for the CT specifications are the same as those described above.

### 2.4 Primary and secondary outcomes and definitions of the outcome measures

Our primary outcome of interest will be reduction in mortality from all causes by upfront use of WBCT in comparison with conventional management (**KQ 4a**). Here, mortality outcomes include 24-h mortality, 30-d mortality, in-hospital mortality, and overall mortality. For all subquestions under **KQ 4**, we will consider the effect of “assignment” to WBCT-based management (vs conventional, selective CT-based management) at baseline (i.e., the ITT-type effect) to be the main analysis; the effect of “starting and adhering” to the WBCT-based management (vs conventional, selective CT-based management) (i.e., the per-protocol-type effect) will be regarded as the sensitivity analysis. We will also assess the effect of WBCT regardless of whether it is performed upfront or as the subsequent-line imaging survey (i.e., as the treated-type effect).

We will assess short-term non-mortality clinical outcomes (**KQs 4b-c**) including time spent before ED disposition for multiple phases of ED stay including time from ED admission to CT, time from ED admission to diagnosis, time from ED admission to specific therapies (e.g., intubation followed by mechanical ventilation, operation, or IVR), time from admission to discharge from ED, time spent for specific therapeutic or supportive interventions, and time spent for ICU or hospital stay by WBCT in comparison with conventional management as the secondary outcomes. Specific clinical characteristics that are associated with better mortality or other short-term clinical outcomes using WBCT (i.e., effect modifiers) will be assessed simultaneously in this context as a secondary outcome (**KQ 5**).

We will also assess clinical outcomes stemming from incidental findings other than trauma, such as cancer or aneurysm, as secondary outcomes (**KQ 6**). These outcomes will include detection of incidental cancers, aneurysms, and other incidental findings. Two other clinical outcomes related to these incidental findings—additional management required for the incidental findings and their subsequent outcomes—will also be assessed as the secondary outcomes (**KQ 7**). Additional management will include specific diagnostic and/or therapeutic interventions deemed necessary (**KQ 7a-1**) and actually performed (**KQ 7a-2**) for the incidental findings. The subsequent clinical outcomes will include any benefits (**KQ 7b-1**) and harms (**KQ 7b-2**) associated with the detection of incidental findings (e.g., reduction in the incidence of [or prevention of the progression of] cancers or aneurysms, detected disease-specific mortality; or, morbidity or mortality due to additional interventions).

Additional immediate outcomes, also assessed as secondary outcomes, include sensitivity and specificity of WBCT to detect traumatic injuries (**KQ 1**), change in the diagnosis of traumatic injuries between WBCT in comparison with conventional imaging-based assessment (**KQ 2**), management decision impact of WBCT, such as additional use of other diagnostic tests or supportive (e.g., blood transfusion, presser, and ventilator) or therapeutic interventions (e.g., surgery and IVRs) ordered based on the WBCT results in comparison with conventional imaging-based assessment (**KQ 3**).

### 2.5 Metrics of outcomes, numerical data to extract, and operational definitions

Sensitivity and specificity will be used as the metric of diagnostic accuracy (**KQ 1**). We will define sensitivity as *TP*/(*TP*+*FN*) and specificity as *TN*/(*FP*+*TN*), where *TP* indicates true-positive (positive WBCT and reference standard tests), *FP* indicates false-positive (WBCT positive and reference standard test negative), *FN* indicates false-negative (WBCT negative and reference standard test positive), and *TN* indicates true-negative (WBCT and reference standard test negative) results from the 2 × 2 contingency table including cross-classified count data according to whether WBCT and the reference standard test are positive or negative.

For sensitivity and specificity, we will focus on (a) injuries that occur in 5 specific ISS-based body regions (i.e., head and neck, face, chest, abdomen, and pelvic ring) separately and (b) 20 operationally defined individual injuries as proposed by Stengel et al. separately (i.e., skull fracture, brain injury, injuries in the carotid or vertebral arteries, or cervical veins; cervical spine injury; face injury; thoracic spine injury; serial rib fractures; lung contusion; pneumothorax; hemothorax; aortic and cardiac injuries; lumbar spine injury; liver injury; splenic injury; hollow visceral and mesenterial tears; hemoperitoneum; retroperitoneal bleeding; kidney injury; pelvic ring fracture; acetabular fracture),^[35]^ and consider an individual patient as the unit of analysis (i.e., patient-based analysis).

We will use the percentage of absolute change in the diagnosis of traumatic injuries in each of the 5 ISS-based body regions or 20 individual injuries between WBCT and conventional imaging-based assessment as the measure of diagnostic impact (**KQ 2**) and again consider an individual patient as the unit of analysis. For diagnostic before-after studies,^[36]^ we will define absolute change in the diagnosis as the number of injured patients (for a specific body region or individual injury), detected by WBCT, subtracted by the number of injured patients detected by a conventional diagnostic strategy (e.g., the primary survey with chest and pelvic X-rays and FAST followed by CT of selected body regions), which is divided by the total number of all patients tested with WBCT.

As the measures of management decision impact outcomes, specifically related to traumatic injuries (**KQ 3**) and incidental findings (**KQ 7a**), we will calculate the absolute change or relative risk of additional diagnostic or therapeutic interventions. The unit of analysis will be an individual patient. For diagnostic before-after studies,^[36]^ we will calculate the number of patients for whom specific diagnostic or therapeutic interventions were added or avoided according to WBCT in comparison with conventional imaging-based assessment, divided by the total number of patients tested with WBCT as the absolute change. For RCTs and NRSIs (typically performed with a controlled before-after design or controlled interrupted time series, where comparisons of the management of patients are made between 2 groups and one before and the other after the introduction of upfront use of WBCT),^[37]^ we will use the relative risk (RR) in a specific intervention between the 2 groups.

For patient-relevant clinical outcomes specifically related to traumatic injuries (**KQ 4**) and incidental findings (**KQ 7b**), we will assess the association of use of upfront WBCT (vs conventional, no-WBCT management) with binary, event-type count data (e.g., all-cause mortality, trauma-specific mortality, cancer- or aneurysm-specific mortality) by using RR or hazard ratio (HR) as the measures of outcomes. For continuous outcomes, such as length of hospital stay and other duration outcomes, we will use the difference in time spent between groups.

For incidental findings (**KQ 6**), we will calculate the percentage of incidental findings as the outcome metric. Our main target condition of interest was incidental findings that were verified as malignancy only. We will also consider composite target conditions—major incidental findings (that may cause mortality) alone, and major and moderate findings combined (that may cause morbidity and mortality) according to the commonly adopted three-grade^[38]^ and four-grade^[39]^ classification systems for incidental CT findings.

### 2.6 Confirmation of outcomes

For sensitivity and specificity (**KQ 1**) and diagnostic impact outcomes (**KQ 2**), we will accept any reference standard tests adopted in eligible studies. However, we will prefer clinical verification including repeat and/or additional imaging, surgical or radiological interventions, and clinical follow-up and autopsy, if performed, as the appropriate clinical reference standard over WBCT (performed as the index test) only. Likewise, we will accept any methods to verify event-type and duration-type clinical outcomes for **KQs 4–6**; however, we will record whether a study prospectively identified the events or durations through research-oriented, study-specific methods (preferred), or relied on data, routinely collected for purposes other than research such as data from disease or trauma registries, or medical charts.^[40]^

### 2.7 Assessment of risk of bias

To assess the risk of bias and concerns regarding the applicability of studies of diagnostic accuracy (**KQ 1**), 2 reviewers will independently assess the patient selection, index test, reference standard, and their flow and timing, based on the revised Quality Assessment of Diagnostic Accuracy Studies instrument tool (QUADAS-2).^[41]^ We will assess 4 domains of study validity: participant selection, index test, reference standard test, and flow and timing thereof. A tailored version of the tool with details of the signaling questions, operational definitions, and rules for combining the answers to produce a domain-level rating are included in the supplementary file, http://links.lww.com/MD/F533.

For diagnostic before-after studies that assessed diagnostic impact (**KQ 2**), we will assess study quality by using the original version of the Quality Assessment of Diagnostic Accuracy Studies instrument tool (QUADAS) tool with modifications proposed by Meads et al.^[42]^ As recommended, we will apply the adapted QUADAS tool by considering conventional imaging-based assessment (i.e., the conventional primary survey followed by focused CT) and WBCT as the (hypothetical) index and reference standard tests, respectively.

To assess patient management and clinical outcomes (**KQs 3, 4,** and **7**), we will use established quality assessment tools. Our main interest is the ITT effect of upfront use of WBCT (i.e., the effect of assignment to an upfront WBCT-based management at baseline) on mortality. Regarding NRSIs, we will use the Risk Of Bias In Non-randomized Studies of Interventions (ROBINS-I tool).^[43]^ We will assess 7 domains of study validity, i.e., confounding, participant selection, classification of interventions, deviations from intended interventions, missing data, measurement of outcomes, and selective reporting. RCTs, we will use the revised tool to assess risk of bias in randomized trials (RoB 2 tool).^[44]^ We will assess 5 domains of trial validity, i.e., randomization processes, deviations from intended interventions, missing outcome data, measurement of outcomes, and selective reporting. We will prespecify age, GCS, SBP, RR, and ISS, and the TRISS and its variants as important mortality predictors and prediction models to consider, respectively.^[45]^

### 2.8 Data synthesis

To quantitatively synthesize sensitivity and specificity (**KQ 1**), we will first calculate sensitivity and specificity for each study with their corresponding 95% confidence intervals (CIs). Then, we will assess between-study heterogeneity visually by plotting sensitivity and specificity separately in forest plots and in the receiver operating characteristic (ROC) space. When studies are less likely to have different explicit thresholds for diagnosing injury findings, we will obtain summary estimates of sensitivity and specificity with their corresponding 95% credible intervals (CrIs) and confidence regions for summary sensitivity and specificity by using bivariate random-effects meta-analysis with the exact binomial likelihood when ≥3 studies are available.^[46,47]^ When diagnostic criteria are evidently inconsistent across studies, we will construct hierarchical summary ROC curves (HSROCs).^[48]^

Use of imperfect reference standards is a well-recognized problem that can preclude accurate estimation of sensitivity and specificity of a test of interest (i.e., index test) even when a series of clinical tests are performed to verify a disease condition (typically, all the test results are combined and assessed based on the “OR” rule—patients with ≥1 positive test are classified as disease positive).^[49]^ Direction and extent of bias in the estimation of sensitivity and specificity of an index test depend on the sensitivity and specificity of the reference standards and conditional dependence or independence on the true disease status between the index and reference standard tests.^[50]^ To address the effect of imperfect reference standards, we will perform a latent-class model meta-analysis with noninformative priors as sensitivity analysis.^[51]^

For other (non-accuracy) outcomes, we will assess between-study heterogeneity visually by constructing forest plots and quantitatively by estimating the between-study SD parameter, *tau*, and *I*^*2*^ statistics and their corresponding 95% CrIs. An *I*^*2*^ > 50% will indicate intermediate heterogeneity, while an *I*^*2*^ > 70% will indicate high heterogeneity.^[52]^ To additionally explore statistical heterogeneity in studies of patient management and patient-relevant clinical outcomes (**KQ 3, 4, and 7**), we will identify influential and/or outlier studies by constructing the Graphical Display of Study Heterogeneity (GOSH) plots if data are amenable to this analysis.^[53]^

For calculating summary RR or HR estimates, based on event-type data (**KQ 4, 5, and 7b**), we will perform a standard Bayesian hierarchical random-effects meta-analysis.^[54]^ For count data from RCTs, we will perform a random-effects meta-analysis using the binomial likelihood with logit or complementary log-log link in a generalized linear modelling framework.^[54]^ If already-estimated relative measures are the only extractable formats for RCTs, and for all other designs of comparative studies (i.e., non-randomized studies of interventions), we will use the log-transformed estimates and their variances as “plug-in” estimates and perform a random-effects meta-analysis using the standard approximate normal-normal model. Regarding prior distributions on the *tau*, we will use the context-specific informative prior distributions as appropriate.^[55]^ We will prefer data adjusted for cofounding variables over unadjusted data. For difference in continuous outcomes, such as length of hospital stay, we will perform a random-effects meta-analysis of the standardized mean difference.^[56]^ Again, we will prefer adjusted data over unadjusted data.

For summary estimates of the proportion measures in non-comparative studies (**KQ 2, 3, and 7a**), such as proportions of detection of incidental findings, we will perform a random-effects meta-analysis of proportions using the binomial likelihood and logit link (i.e., the so-called binomial-normal model).^[57]^ We will use a non-informative prior distribution on the *tau* to cover the possibility of very large across-study statistical heterogeneity.

### 2.9 Additional analyses

For studies relevant to each **KQ** (other than **KQ 1)**, we will perform the funnel-plot asymmetry test if at least 10 studies are included.^[58]^ For studies assessing diagnostic accuracy (**KQ 1**), we will not assess funnel-plot asymmetry because the required tests are still less well-established and not recommended for studies of diagnostic accuracy.^[59]^

We will perform subgroup analyses and univariable random-effects meta-regressions, if appropriate.^[54]^ Preplanned candidate factors will include design (e.g., RCT vs NRSI), number of centers (single- vs multi-center), study location, CT protocol, cause of trauma (road traffic injuries vs falls), and severity of included patients (multiple trauma vs not; and AIS > 3 vs not by anatomical regions). For the univariable meta-regression of diagnostic accuracy, we will add a covariate in the bivariate model to simultaneously explain the statistical heterogeneity in both (logit-transformed) sensitivity and specificity.^[60]^

For missing binary outcome data in RCTs (**KQ 4 and 7b**), if feasible, we will apply the Bayesian approach proposed by Turner et al to fully account for uncertainty derived from missing data.^[61]^ Otherwise, we will impute missing data based on a series of conventional methods proposed by Akl et al.^[62]^ For missing continuous data, we will impute missing data as proposed by Ebrahim et al.^[63]^

Finally, we will assess the certainty of evidence using the Grading of Recommendations Assessment, Development, and Evaluation approach.^[64]^ For **KQs** on patient-relevant clinical outcomes (**KQ 4 and 7b**), we will create a standard summary of findings (SoF) tables and assess the overall quality of evidence according to the recommendations.^[65–67]^ For diagnostic accuracy (**KQ 1**), SoF tables and the overall quality of evidence will be constructed following the approaches specifically designed for test accuracy.^[68,69]^

### 2.10 Statistical software

All statistical analyses will be performed in the Bayesian framework using OpenBUGS V.3.2.3 (members of OpenBUGS Project Management Group; see www.openbugs.net) from within Stata V.16.1/SE (Stata Corp. College Station, TX, USA) via a suite of “wbs” commands.^[70]^ All tests will be two-sided, and statistical significance will be defined as a *P* value <.05.

### 2.11 Ethics and dissemination

Ethics review will not be necessary as this is a systematic review of already published data. The review findings will be disseminated through publications in peer-reviewed journals, and presentations at conferences.

## 3 Discussion

Upfront use of WBCT is an alternative mode of trauma screening strategy that is becoming increasingly available. WBCT may become more accessible in the near future as more institutions install a modern, high-quality and high-speed CT scanner system, adjacent to or in the ED, which may ultimately increase the number of trauma patients screened with routine, upfront WBCT. The theoretical advantages of WBCT-based management over selective CT-based management have only been shown for intermediate outcomes, such as time required to diagnose traumas and have yet to be convincingly demonstrated for morbidity and mortality, and more importantly, for patient-relevant clinical outcomes in RCTs. Evidence is lacking on the benefits and harms stemming from incidental findings detected by WBCT. In theory, by expanding the scan targets from selected body parts to the whole body, pan-scanning could detect more injured lesions at the cost of more incidental findings, in addition to the already demonstrated increased overall radiation exposure and cost.^[23]^ Given the immediate cost of additional tests and clinical follow-up for the incidental findings, effectiveness and harms of WBCT-based management *vs.* alternatives, conventional management should be comprehensively assessed as the whole management. With up-to-date systematic review methodologies, and if feasible, new meta-analytic results, we hope to clarify the actionable evidence that supports the evidence-based trauma care using upfront WBCT.

### 3.1 Strengths and limitations of this study

- This will be the first systematic review and meta-analysis to comprehensively assess the existing evidence on diagnosis and subsequent clinical outcomes—including incidental findings—stemming from routine whole-body computed tomography for blunt polytrauma, using the established frameworks to assess diagnostic technology and its health outcomes.
- Comprehensive literature search and up-to-date systematic review methodologies will be used to clarify the strengths and limitations of the currently existing evidence.
- Meta-analyses might not be feasible if appropriately analyzed comparative data were sparse.

## Author contributions

KN, MI, and TT originated the idea; KN and TT performed literature searches and extracted data for protocol writing; KN and TT drafted the initial version of the protocol; all authors suggested amendments and approved the final version of the protocol; all authors made substantial contributions to the intellectual content of the paper and gave final approval for the final version of the manuscript. KN and TT are guarantors of the research.

**Conceptualization:** Kyohei Nagasawa, Mitsunaga Iwata, Teruhiko Terasawa.

**Data curation:** Kyohei Nagasawa, Teruhiko Terasawa.

**Formal analysis:** Teruhiko Terasawa.

**Funding acquisition:** Teruhiko Terasawa.

**Investigation:** Kyohei Nagasawa, Teruhiko Terasawa.

**Methodology:** Kyohei Nagasawa, Mitsunaga Iwata, Takashi Nihashi, Teruhiko Terasawa.

**Project administration:** Mitsunaga Iwata, Takashi Nihashi, Teruhiko Terasawa.

**Software:** Teruhiko Terasawa.

**Supervision:** Mitsunaga Iwata, Takashi Nihashi, Teruhiko Terasawa.

**Validation:** Kyohei Nagasawa, Mitsunaga Iwata, Takashi Nihashi, Teruhiko Terasawa.

**Writing – original draft:** Kyohei Nagasawa, Teruhiko Terasawa.

**Writing – review & editing:** Kyohei Nagasawa, Mitsunaga Iwata, Takashi Nihashi, Teruhiko Terasawa.

## Abbreviations

Abbreviations: AIS = Abbreviated Injury Scale, CT = computed tomography, ED = emergency department, FAST = focused assessment with sonography for trauma, GCS = Glasgow Coma Scale, ISS = Injury Severity Score, ITT = intention-to-treat, IVR = interventional radiology, KQ = key question, NRSI = non-randomized studies of intervention, RCT = randomized controlled trial, RR = respiratory rate, SBP = systolic blood pressure, TRISS = trauma and injury severity score, WBCT = whole-body computed tomography.
